# The effects of camel milk in systemic inflammation and oxidative stress of cigarette smoke-induced chronic obstructive pulmonary disease model in rat

**DOI:** 10.3389/fvets.2024.1464432

**Published:** 2024-12-13

**Authors:** Sepide Behrouz, Mahla Mohammadi, Hadi Sarir, Mohammad Hossein Boskabady

**Affiliations:** ^1^Applied Biomedical Research Center, Mashhad University of Medical Sciences, Mashhad, Iran; ^2^Department of Animal Science, Faculty of Agriculture, University of Birjand, Birjand, Iran; ^3^Department of Physiology, Faculty of Medicine, Mashhad University of Medical Sciences, Mashhad, Iran

**Keywords:** camel milk, COPD, cytokine, oxidative stress, pulmonary disease

## Abstract

**Background:**

The effects of camel milk in inflammation and systemic oxidative stress of cigarette smoke (CS)-induced chronic obstructive pulmonary disease (COPD) associated with small airway inflammation in rats were investigated.

**Methods:**

35 male Wistar rats were randomly divided into five groups: (a) control, (b) CS-exposed rats, c and (d) CS-exposed rats treated with the 4 and 8 mL/kg camel milk, and (e) CS-exposed rats treated with 1 mg/kg dexamethasone.

**Results:**

Total and differential WBC counts, serum level of TNF-*α* and malondialdehyde (MDA) level in serum and homogenized tissues of the heart, kidney, liver, and testicle were significantly increased, but catalase (CAT), superoxide dismutase (SOD) and thiol levels were significantly decreased in CS-exposed rats (*p* < 0.01 to *p* < 0.001). Treatment with dexamethasone and both doses of camel milk improved all measured variables compared to the COPD group (*p* < 0.05 to *p* < 0.001). The improvements of most variables in the treated group with high dose of camel milk were higher than the effect of dexamethasone (*p* < 0.05 to *p* < 0.001). These findings suggest that camel milk has a therapeutic potential for treating systemic oxidative stress and inflammatory induced by CS.

**Conclusion:**

Therefore, camel milk might be effective in attenuating the effects of CS-induced systemic inflammation and oxidative stress.

## Introduction

Chronic obstructive pulmonary disease (COPD) is one of the incurable and common lung diseases that can turn into diseases such as cardiopulmonary disease and respiratory failure (1). Cough, shortness of breath, and increased sputum along with slow development of airflow limitation are the characteristics of this disease. In patients with COPD, increased anxiety and depression, weight loss, anorexia, sleep disorders, and daytime sleepiness were also observed (2). The history of COPD varies among individuals with different COPD phenotypes according to the onset, early stages, and progression of the disease (3). Genetics and environment are two critical factors in the development of COPD as well as other influenced factors include age, gender (4), particle exposure, lung development and growth, socioeconomic status, asthma, infections, and chronic bronchitis (1).

The characteristics of COPD are lung emphysema, fibrosis, and chronic inflammation of small airways (5, 6). COPD may not resolve even with cessation of exposure to cigarette smoke (CS) in the presence of some endogenous factors such as persistent infection or autoimmunity (6). The development and progression of pathogenic mechanisms involved in COPD can occur due to oxidative stress. So exogenous oxidants such as CS and air pollution, endogenous oxidants such as superoxide anions and mitochondrial oxidants, and the reduction of antioxidants such as superoxide dismutase (SOD) and thioredoxin can increase lung oxidant stress in COPD (6).

For physiological cell function, the control of reactive oxygen species (ROS) production is necessary, because an excessive increase in the level of ROS compared to the antioxidant capacity of a cell leads to oxidative stress. Antioxidant enzymes including SOD, catalase (CAT), and glutathione peroxidase (Gpx) neutralize ROS inside cells (7). Thus, considering the influential role of oxidative stress in the pathogenesis of COPD, basic mechanisms in neutralizing oxidative stress are essential and necessary for a more effective treatment of COPD (8).

High concentrations of bioactive compounds including lactoferrin and various immunoglobulins (IgG, IgA, IgM, IgD) are found in camel milk, which has turned camel milk into a substance with extraordinary medicinal properties (9). The presence of high levels of these compounds along with various vitamins (C, B1, B2, E, A) (10), lysozymes, insulin-like molecules and lactoperoxidase cause the therapeutic potential of camel milk in many diseases such as asthma, hepatitis, autism, cancer, diabetes, jaundice, tuberculosis, food allergies and diarrhea. Therefore, the high concentrations of whey proteins, lactoferrin, casein, lactic acid bacteria (LAB) and vitamin C in camel milk may be the main factor in reducing oxidative stress and thus its antioxidant effects (11). The expression of the genes carried out by the peptides isolated from camel milk has increased significantly, indicating the clearing of free radicals and antioxidant properties of camel milk and as a result neutralization of stress and apoptosis (12).

Therefore, in the present study, the effects of camel milk on systemic inflammation and oxidative stress of CS-induced COPD in rats were investigated.

## Materials and methods

### Animal groups and the CS-induced COPD protocol

From the animal house of Mashhad University of Medical Sciences, 35 Wistar rats (male, 200 ± 250 g) were purchased and kept in standard conditions (free access to food and water, 12 h light/dark cycle, 22 ± 2°C and humidity 54 ± 2%). This study was approved by the ethics committee of Mashhad University of Medical Sciences in animal experiments (Code 981778).

The animals were randomly allocated to the following groups (n = 7 in each group):

(A) Control (Ctrl) group
(B) CS-exposed (COPD) group
(C) CS-exposed and treated with the 4 mL/kg camel milk (CM-L) group (13).
(D) CS-exposed and treated with the 8 mL/kg of camel milk (CM-H) group (13)
(E) CS-exposed and treated with 1 mg/kg dexamethasone (Dexa) group (13).

**Figure 1:**
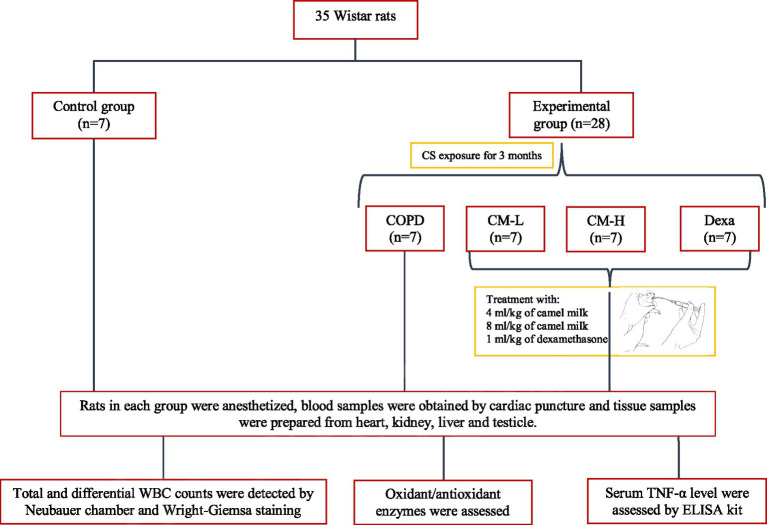
Flowchart of the study design of the animal model.

Based on the model by Mehtaj et al. (14), rats were exposed to the CS for three consecutive months, except the control group. To acclimate the rats to the CS, the number of cigarettes was gradually increased over the first 4 days: from one cigarette on the first day to two on the fifth day, three on the ninth day, four on the thirteenth day, and five cigarettes on the seventeenth day. Following this 20-days initial period, each rat was then exposed to five cigarettes per week for the remaining duration of the 3 months (13, 14), (Figure 1).

### Preparation of camel milk

Camel milk was purchased from the Asaish Company in Gonbad kavos, Iran. The camel’s milk in the Turkmen Sahara region contains 2.9% protein, 83–90% water, 4.52% lactose, 12.38% total solids, 4.19% fat, and 0.77% ash (15). Using the lyophilization method (16), camel milk was prepared in powder form and mixed with drinking water (0.2 g/mL). Different concentrations of the resulting solution (4 and 8 mL/kg) were administered to rats by gavage.

### Blood collection, measurement of total and differential WBC and biochemical analysis

Rats were anesthetized by intraperitoneal injection of 50 mg/kg ketamine at the end of the experimental period (3 months) and sacrificed and 5 mL blood was taken from the heart. Then, 1 ml of the blood was added to a coagulated tube for counting white blood cells (WBC) and 4 mL was centrifuged at 3000 rpm for 15 min and the serum was stored at 4°C for measuring oxidative stress markers and cytokines (17).

The total and differential number of WBC were measured using a Neubauer chamber and Wright-Giemsa staining of a smear of the blood, respectively, (18).

The level of SOD, CAT, thiol and malondialdehyde (MDA) were measured in the serum, homogenized tissues of the heart, kidney, liver, and testicle tissues based on previous studies. SOD activity was assessed by measuring superoxide generation through the auto-oxidation of pyrogallol, and its activity was indirectly evaluated at 570 nm (19). The rate at which hydrogen peroxide (H_2_O_2_) decomposes by CAT was assessed using Aebi’s method, and its activity was measured with a spectrophotometer at 240 nm (20). Total thiol content was assessed by generating a yellow complex from SH groups, which exhibits an absorbance peak at 412 nm (21). The reaction of MDA with thiobarbituric acid (TBA) results in a pink solution, which has an absorbance peak at 535 nm (22). Also, using a double enzyme-linked immunosorbent assay (ELISA) kit (Carmania Pars, Kerman, Iran), the concentration of tumor necrosis factor-alpha (TNF-*α*) was measured in the serum (23, 24).

### Statistical analysis

The mean ± standard error of the mean (SEM) of the data was presented. Statistical data comparison was done using one-way analysis of variance (ANOVA) with Tukey–Kramer post-test using Instat software and *p* < 0.05 was considered as significance criteria.

## Results

### Effect of camel milk on total and differential WBC count in the serum

**Figure 2:**
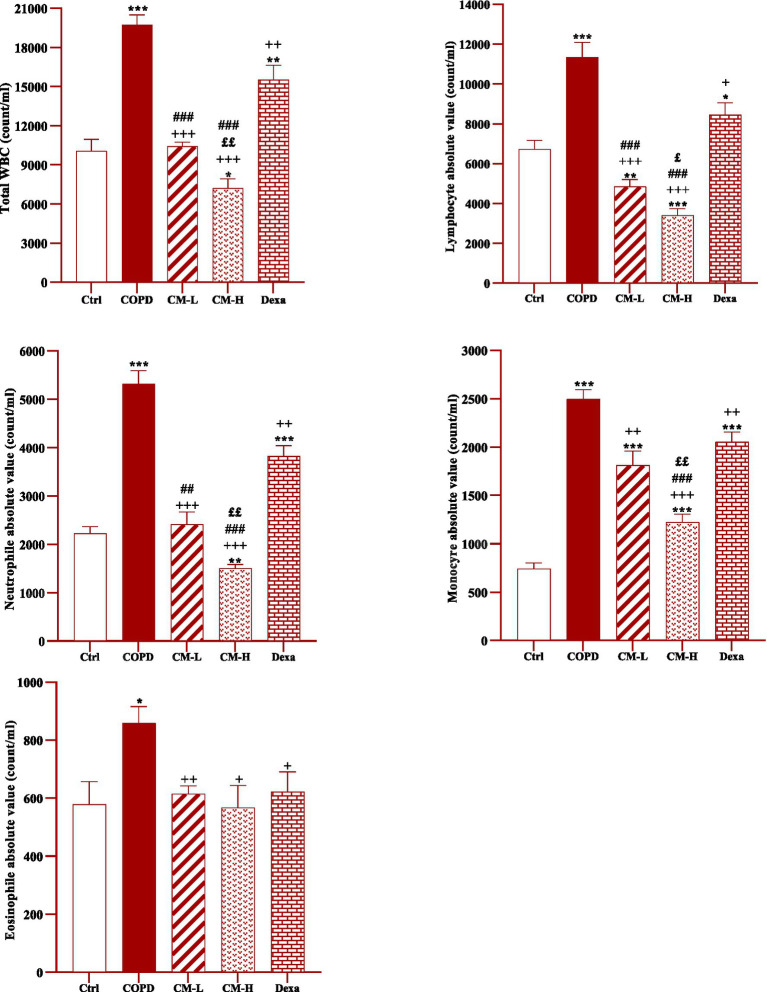
Effects of camel milk on improving total and differential WBC counts in the blood of CS-exposed rats. Data are presented as mean ± SEM (*n* = 7 in each group). *p < 0.05, **p < 0.01 and ***p < 0.001; compared to the control group. +*p* < 0.05, ++*p* < 0.01 and +++*p* < 0.001; compared to untreated COPD group. £*p* < 0.05 and ££*p* < 0.01; compared to COPD group-treated with 4 mL/kg camel milk. ##*p* < 0.01 and ###*p* < 0.001; compared to Dexa group. ANOVA and Tukey–Kramer test were used for statistical analysis.

In CS-exposed rats, total and differential WBC, neutrophil, monocyte, lymphocyte and eosinophil counts in the blood were significantly increased compared to the control rats (*p* < 0.05 for eosinophil and *p* < 0.001 for other cases, Figure 2).

Total and differential WBC counts in the blood in CS-exposed rats treated with two doses of camel milk and dexamethasone were significantly reduced compared to the COPD group (*p* < 0.05 to *p* < 0.001, Figure 2).

The effects of a high dose of camel milk on total and differential WBC were higher than its low dose except for eosinophil (*p* < 0.05 for lymphocyte and *p* < 0.01 for other cases, Figure 2). In CS-expo rats treated with two doses of camel milk total and differential WBC were lower than the dexamethasone treated group except monocyte in those treated with a lower dose and eosinophil in those treated with both doses of camel milk (p < 0.05 to *p* < 0.001, Figure 2).

In CS-exposed rats treated with a high dose of camel milk, total and differential WBC were lower than its low dose except for eosinophil (*p* < 0.05 to *p* < 0.01, Figure 2).

### Effect of camel milk on TNF-*α* level in the serum

**Figure 3:**
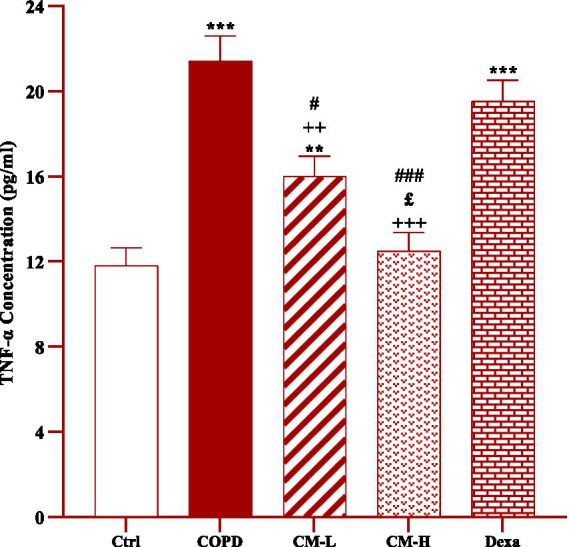
Effect of camel milk on improving TNF-α level in serum of CS-exposed rats. Data are presented as mean ± SEM (*n* = 7 in each group). **p < 0.01 and ***p < 0.001; compared to the control group. ++*p* < 0.01 and +++*p* < 0.001; compared to untreated COPD group. £*p* < 0.05; compared to COPD group-treated with 4 mL/kg camel milk. #*p* < 0.05 and ###*p* < 0.001; compared to Dexa group. ANOVA and Tukey–Kramer test were used for statistical analysis.

The level of TNF-α in the serum of CS-exposed rats was significantly increased compared to the control rats (*p* < 0.001, Figure 3).

The serum level of TNF-*α* in CS-exposed rats treated with two doses of camel milk was significantly reduced compared to the non-treated CS-exposed rats (*p* < 0.01 for low dose and *p* < 0.001 for high dose of camel milk, Figure 3). In the CS-exposed rats treated with a low dose of camel milk and dexamethasone, the level of TNF-α in the serum was lower than in the control rats (*p* < 0.01 for a low dose of camel milk and *p* < 0.001 for dexamethasone, Figure 3).

The level of TNF-α in the serum of CS-exposed rats treated with a high dose of camel milk was significantly lower than its low dose (*p* < 0.05, Figure 3).

The effects of both doses of camel milk on serum TNF-α level were significantly higher than dexamethasone (*p* < 0.05 and *p* < 0.001 for low and high dose of camel milk respectively, Figure 3).

### Effect of camel milk on oxidative stress markers

**Figure 4:**
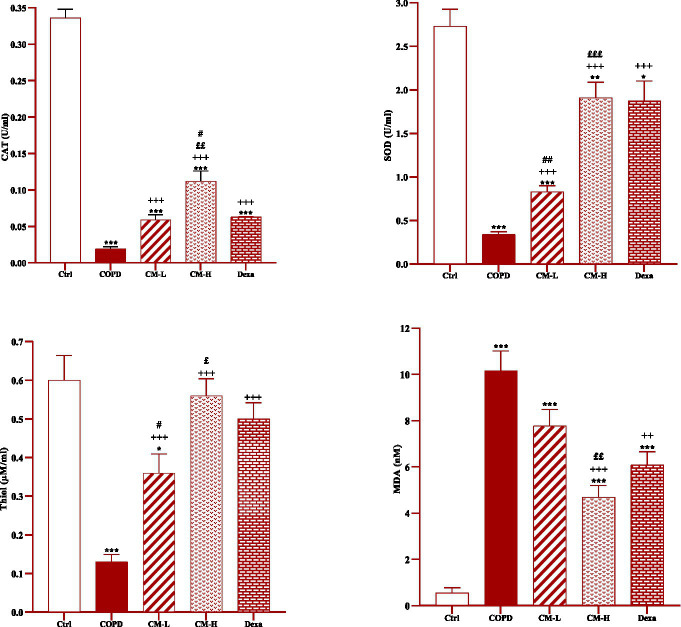
Effects of camel milk on improving CAT, SOD, thiol and MDA levels in serum of CS-exposed rats. Data are presented as mean ± SEM (*n* = 7 in each group). *p < 0.05, **p < 0.01 and ***p < 0.001; compared to the control group. ++*p* < 0.01 and +++*p* < 0.001; compared to untreated COPD group. £*p* < 0.05, ££*p* < 0.01 and £££*p* < 0.001; compared to COPD group-treated with 4 mL/kg camel milk. #*p* < 0.05 and ##*p* < 0.01; compared to Dexa group. ANOVA and Tukey–Kramer test were used for statistical analysis.

**Figure 5:**
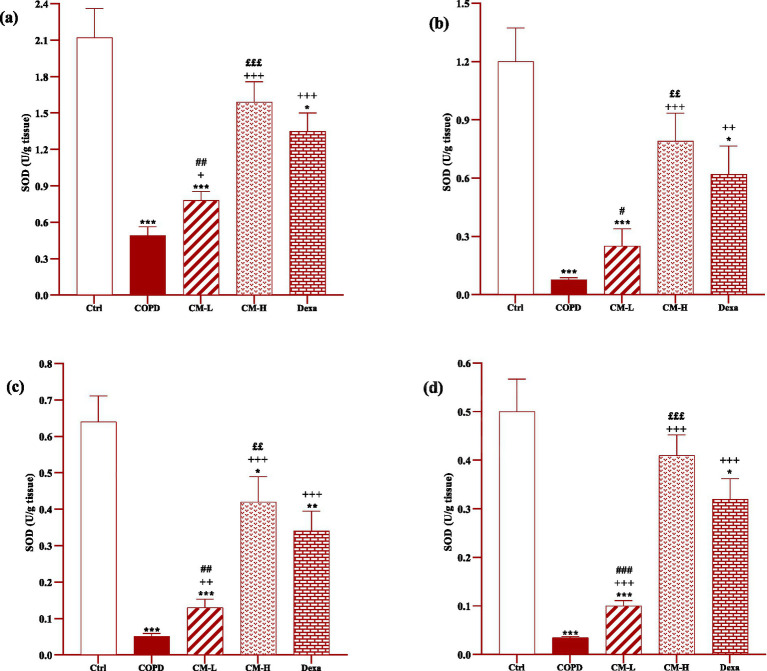
Effects of camel milk on improving SOD levels in the heart **(A)**, kidney **(B)**, liver **(C)**, and testicle tissues **(D)** of CS-exposed rats. Data are presented as mean ± SEM (*n* = 7 in each group). *p < 0.05, **p < 0.01 and ***p < 0.001; compared to the control group. +*p* < 0.05, ++*p* < 0.01 and +++*p* < 0.001; compared to untreated COPD group. ££*p* < 0.01 and £££*p* < 0.001; compared to COPD group-treated with 4 mL/kg camel milk. #*p* < 0.05, ##*p* < 0.01 and ###*p* < 0.001; compared to Dexa group. ANOVA and Tukey–Kramer test were used for statistical analysis.

**Figure 6:**
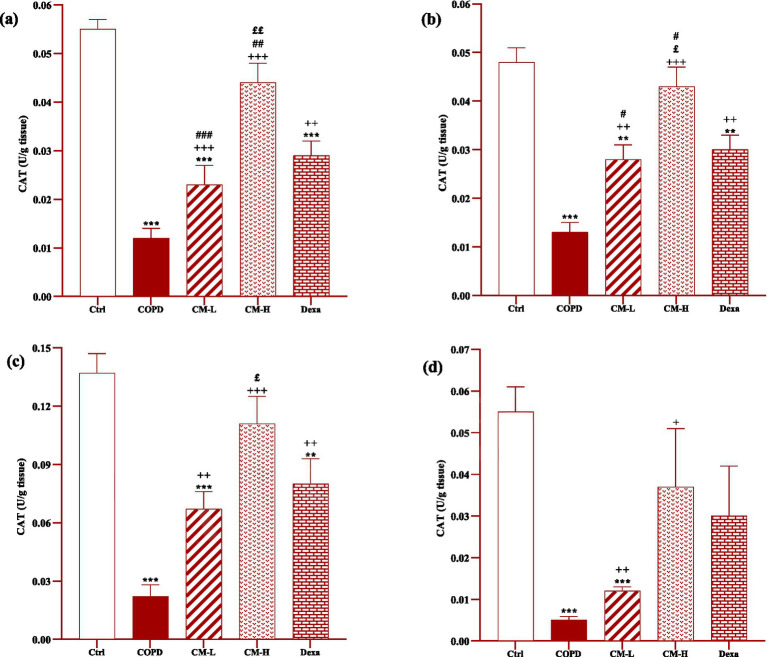
Effects of camel milk on improving CAT levels in the heart **(A)**, kidney **(B)**, liver **(C)**, and testicle **(D)** tissues of CS-exposed rats. Data are presented as mean ± SEM (*n* = 7 in each group). **p < 0.01 and ***p < 0.001; compared to the control group. +*p* < 0.05, ++*p* < 0.01 and +++*p* < 0.001; compared to untreated COPD group. £*p* < 0.05 and ££*p* < 0.01; compared to COPD group-treated with 4 mL/kg camel milk. #*p* < 0.05, ##*p* < 0.01 and ###*p* < 0.001; compared to Dexa group. ANOVA and Tukey–Kramer test were used for statistical analysis.

**Figure 7:**
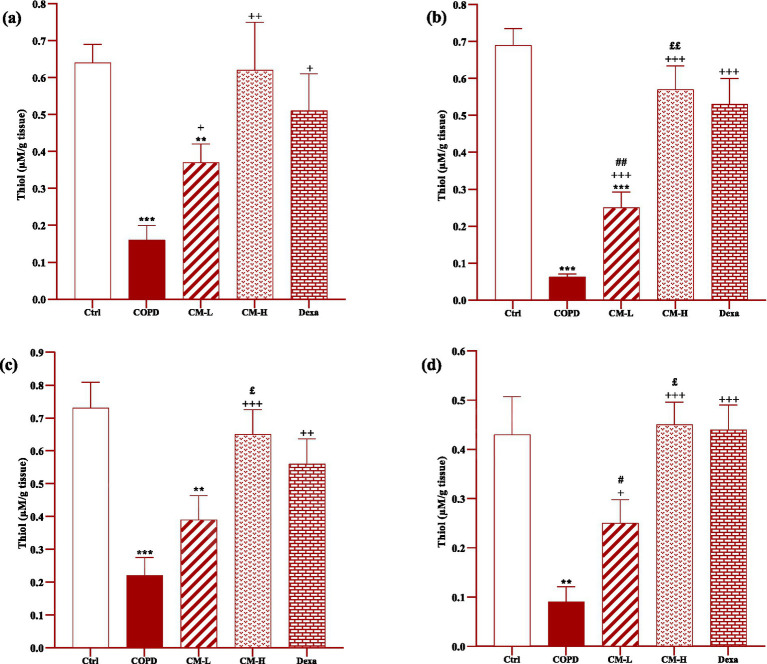
Effects of camel milk on improving thiol levels in the heart **(A)**, kidney **(B)**, liver **(C)**, and testicle **(D)** tissues of CS-exposed rats. Data are presented as mean ± SEM (*n* = 7 in each group). **p < 0.01 and ***p < 0.001; compared to the control group. +*p* < 0.05, ++*p* < 0.01 and +++*p* < 0.001; compared to untreated COPD group. £*p* < 0.05 and ££*p* < 0.01; compared to COPD group-treated with 4 mL/kg camel milk. #*p* < 0.05 and ##*p* < 0.01; compared to Dexa group. ANOVA and Tukey–Kramer test were used for statistical analysis.

**Figure 8:**
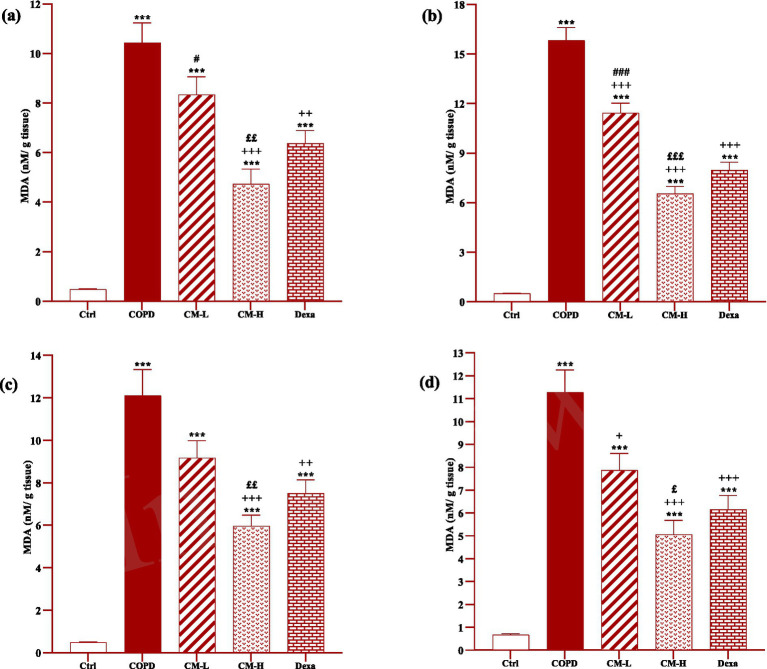
Effects of camel milk on improving MDA levels in the heart **(A)**, kidney **(B)**, liver **(C)**, and testicle **(D)** tissues of CS-exposed rats. Data are presented as mean ± SEM (*n* = 7 in each group). ***p < 0.001; compared to the control group. +*p* < 0.05, ++*p* < 0.01 and +++*p* < 0.001; compared to untreated COPD group. £*p* < 0.05, ££*p* < 0.01 and £££*p* < 0.001; compared to COPD group-treated with 4 mL/kg camel milk. #*p* < 0.05 and ###*p* < 0.001; compared to Dexa group. ANOVA and Tukey–Kramer test were used for statistical analysis.

In the serum and homogenized tissues of the heart, kidney, liver, and testicle, SOD and CAT activities and thiol level in the CS-exposed rats were significantly decreased but the MDA level was significantly increased compared to the control group (*p* < 0.001 in all cases, Figures 4–8).

In CS-exposed rats treated with two doses of camel milk and dexamethasone, in the serum, homogenized heart, kidney, liver, and testicle tissues, SOD and CAT activities and thiol level were significantly increased but the MDA level was significantly decreased compared to the non-treated CS-exposed rats except some cases in the treated group with a low dose of camel milk and only one case in the dexamethasone treated group (*p* < 0.05 to *p* < 0.001, Figures 4–8). In CS-exposed rats treated with two doses of camel milk and dexamethasone, oxidative stress markers the improvement in some cases were significantly lower compared to the control rats (*p* < 0.05 to *p* < 0.001, Figures 4–8).

The improvement in oxidative stress markers in the serum, and homogenized heart, kidney, liver and testicle tissues in the treated group with a high dose of camel milk, in most cases were higher than its low dose and, in some cases, than dexamethasone. However, the effects of a low dose of camel milk on some cases of oxidative stress markers were lower than dexamethasone (*p* < 0.05 to *p* < 0.001, Figures 4–8).

## Discussion

In this study, rats were exposed to CS for three months to induce an experimental model of COPD and the effect of treatment with two doses of camel milk and dexamethasone during the exposure period to CS was examined. The findings revealed increased total and differential WBC and TNF-*α* level in the serum and level of MDA in the serum and homogenized tissues of heart, kidney, liver, and testicle, but decreased levels of CAT, SOD and thiol in the COPD group. In all experimental groups except the control group, exposure to cigarettes resulted in weight loss, a persistent cough, a fatigued appearance, decreased mobility, inadequate feeding, dull fur, yellowish urine, and dry stools. However, these symptoms showed improvement in the treatment groups. In addition, the same methodology for induction of COPD was applied in our previous studies (18, 25). Also, in another study we investigated the lung pathological changes caused by cigarette smoke in a rat model of COPD which indicated that cigarette smoke effectively induces COPD in rats (13).

Camel milk (4 and 8 mL/kg) and dexamethasone reduced total and differential WBC counts in the blood, the serum level of TNF-α, and the level of MDA in the serum and homogenized tissues of the heart, kidney, liver, and testicle, but significantly increased the levels of antioxidant enzymes and thiol level.

In the peripheral blood of COPD patients, an increase in the number of white blood cells and systemic inflammation have been reported (26). Smoking can influence the correlation between WBC count and lung function. One of the potential markers in COPD patients is WBC count, which can predict the severity of COPD together with the chest examination and lung function tests (27). The other clinical indicators of acute exacerbation of COPD is blood eosinophil count. Considering the activation of various immune cells including monocytes and lymphocytes in the lungs and the prominent role of neutrophils in the pathogenesis of inflammation, the counting of differential WBC is an indicator of COPD severity (27, 28). These evidences support the changes in rats exposed to CS in this study.

Camel milk is a potent protector against cyclophosphamide (CYP)-induced toxicity that prevents structural changes in leukocytes of leukopenia rats (29). Camel milk is a rich source of biological proteins, iron and B vitamins, which are essential for red blood cells. As a result, the consumption of camel milk increased the number of red blood cells and the concentration of fetal hemoglobin in sickle cell anemia. So, camel milk can prevent anemia crises and manage sickle cell diseases (30). In mice with immunosuppression caused by CYP, the number of WBC, lymphocyte, and neutrophil were significantly decreased but treatment with camel milk increased the concentration of immunoglobulins and antioxidant enzymes and inhibited oxidative stress (31).

In rats with COPD caused by CS exposure, serum and pulmonary tissue TNF-*α* levels were increased (32, 33). The increase of TNF-*α* in the serum of stable COPD patients can be a biomarker reflecting the systemic inflammatory response (34). The systemic inflammatory response in COPD can be justified through systemic hypoxia, which stimulates the local expression of inflammatory cytokines, including TNF-a and IL-6 (34, 35). Also an increase in the serum TNF-*α* level, which could be associated with bronchial patency disorder in COPD was observed (36). The effect of some cytokines, including TNF-α, on the development of pathophysiological mechanisms in COPD may be due to the correlation between serum level of TNF-α and lung function parameters (37).

In the present study, the serum level of TNF-α significantly decreased due to the administration of camel milk. Since TNF-α is mainly produced by macrophages and it is the main regulator of inflammatory responses, camel milk can inhibit inflammation by reducing TNF-*α*. In a clinical study of 64 children with autism, milk reduced neuro-inflammation and gastrointestinal symptoms (38). Administration of six camel milk LAB strains in rats with lipopolysaccharide/galactosamine-induced acute liver damage, reduced the level of TNF-α in the liver (39).

Increased total and differential WBC and TNF-α level in the serum and MDA levels in the serum and homogenized tissues of the heart, kidney, liver, and testicle, but decreased levels of CAT, SOD and thiol in the COPD group were seen in the present study. Cell damage caused by oxidative stress is inhibited by antioxidants as the most essential defense mechanism, antioxidant enzymes can have potential therapeutic effects against inflammatory diseases, rheumatoid arthritis, cancer and neurological diseases (40). According to previous studies, an increase in the serum level of MDA in COPD patients was reported (41). The results of a double-blind randomized clinical trial showed decreased levels of SOD, CAT and thiol in patients with COPD (42).

Treatment with camel milk, reduced total and differential WBC counts in the blood, the serum levels of TNF-*α*, and the level of MDA in the serum and homogenized tissues of the heart, kidney, liver, and testicle, but increased the levels of antioxidant enzymes and thiol level in the present study. The activities of SOD and CAT in rats poisoned with ammonium chloride were significantly increased by camel milk. Camel milk is rich in zinc, magnesium, vitamins C, A, B2 and E (43), which can protect against cadmium-induced anemia by reducing the production of free radicals in red blood cells (44). High levels of α-lactalbumin, *β*-casein and vitamin C have made camel milk an antioxidant that can prevent the production of superoxide anions, free radicals and ROS (11). Camel milk protein hydrolysates can increase SOD and CAT levels in streptozotocin-induced diabetic rats and decrease MDA level. Camel milk with antioxidant properties, improves the symptoms of hyperglycemia and hyperlipidemia (45). Due to the immune system strengthening and anti-toxic properties of camel milk, in mice with leukopenia caused by CYP, hepatic SOD and CAT were increased compared to the untreated group (29).

In the present study, the pulmonary function tests of experimental groups and oxygen saturation results were not provided which should be performed in further studies. Also, it is suggested that in future studies a third group treated with higher camel milk be investigated to drive a causal regression analysis. The camel milk may contain residue-free, especially about the chemicals affecting pulmonary function or the studied parameters. However, the results of the current study indicated beneficial effects on all measured parameters. Considering the presence of residue-free, affecting pulmonary function or the studied parameters in the camel milk, the measured parameters should be deteriorated or at least do not improved. In away in further studies, the appropriate methods should be taken to ensure regarding residue-free, of the camel milk.

The results showed systemic antioxidant and anti-inflammatory properties of camel milk in a rats model of COPD caused by CS exposure, which are more effective compared to dexamethasone. Therefore, camel milk could be considered for the treatment of inflammatory diseases including asthma and COPD.

## Data Availability

The raw data supporting the conclusions of this article will be made available by the authors, without undue reservation.
